# Mechanisms of SARS-CoV-2 Infection-Induced Kidney Injury: A Literature Review

**DOI:** 10.3389/fcimb.2022.838213

**Published:** 2022-06-14

**Authors:** Weihang He, Xiaoqiang Liu, Bing Hu, Dongshui Li, Luyao Chen, Yu Li, Yechao Tu, Situ Xiong, Gongxian Wang, Jun Deng, Bin Fu

**Affiliations:** ^1^ Reproductive Medicine Center, The First Affiliated Hospital of Nanchang University, Nanchang, China; ^2^ Department of Urology, The First Affiliated Hospital of Nanchang University, Nanchang, China; ^3^ Jiangxi Institute of Urology, Nanchang, China

**Keywords:** SARS-CoV-2, COVID-19, ACE2, Acute kidney injury, TMPRSS2

## Abstract

The severe acute respiratory coronavirus 2 (SARS-CoV-2) has become a life-threatening pandemic. Clinical evidence suggests that kidney involvement is common and might lead to mild proteinuria and even advanced acute kidney injury (AKI). Moreover, AKI caused by coronavirus disease 2019 (COVID-19) has been reported in several countries and regions, resulting in high patient mortality. COVID-19‐induced kidney injury is affected by several factors including direct kidney injury mediated by the combination of virus and angiotensin-converting enzyme 2, immune response dysregulation, cytokine storm driven by SARS-CoV-2 infection, organ interactions, hypercoagulable state, and endothelial dysfunction. In this review, we summarized the mechanism of AKI caused by SARS-CoV-2 infection through literature search and analysis.

## Introduction

Coronavirus disease 2019 (COVID-19), caused by severe acute respiratory syndrome coronavirus 2 (SARS-CoV-2) infection, has rapidly developed into a global pandemic. By February 18, 2022, 420 million confirmed cases and 5,884,120 deaths have been recorded worldwide (https://www.worldometers.info/coronavirus/). The clinical manifestations of the disease range from mild respiratory disease to severe progressive pneumonia and multiple organ dysfunction (Naicker et al., 2020). SARS-CoV-2 binds to angiotensin-converting enzyme 2 (ACE2) and then its spike protein is hydrolyzed and cleaved by type II transmembrane serine protease (TMPRSS2), thereby fusing the virus and cell membrane and invading the cells (Hoffmann et al., 2020; Matsuyama et al., 2020). The Human Protein Atlas database shows that the expression of ACE2 and TMPRSS2 genes was comparable in kidney and lung tissue (Pan et al., 2020). Recent studies have shown that SARS-CoV-2 mainly invades podocytes and proximal tubule cells of the kidney (Diao et al., 2021a) and the presence of intracellular viral arrays in proximal tubular epithelial cells has been discovered by electron microscopy. Moreover, clinical evidence suggests that kidney involvement is common and can lead to acute kidney injury (AKI) and even high mortality (Ronco et al., 2020). We performed a systematic search in PubMed to identify recently published large cohort studies related to AKI in patients with COVID-19. We used the search terms “Coronavirus”, “COVID-19”, “SARS-CoV-2”, “acute kidney damage”, “acute kidney injury”, “AKI” and found that the incidence of AKI was higher in severe COVID-19 patients and AKI was strongly associated with high mortality (**Table 1**) (Bell et al., 2021; Chen et al., 2021; Jewell et al., 2021; Kilis‐pstrusinska et al., 2021; Marques et al., 2021; Procaccini et al., 2021; Rahimzadeh et al., 2021; Scarpioni et al., 2021; Sullivan et al., 2021; Strohbehn et al., 2021; Wan et al., 2021; Hung et al., 2022; Hsu et al., 2022; Sindhu et al., 2022). Many possible mechanisms for COVID-19-induced kidney injury have been proposed, including the direct kidney injury mediated by the combination of virus and ACE2. The immune response dysregulation driven by SARS-CoV-2, including cytokine storm and lymphopenia, may indirectly affect the kidneys and organ interactions, such as between the lungs, heart, and kidneys, result in hypoxic delivery to the kidneys and may lead to ischemic injury. In addition, the hypercoagulable state caused by SARS-CoV-2 infection and the application of nephrotoxic drugs are potential causes of kidney injury (Ahmadian et al., 2021; Akilesh et al., 2021). This review summarizes the mechanism by which kidney injury is induced in patients with COVID-19 and it helps to elucidate the pathogenic mechanisms of kidney injury caused by SARS-CoV-2, so as to design better therapeutic strategies.

**Table 1:** Data were extracted from 14 large cohort studies of patients with COVID-19, including total number of patients included, incidence of AKI, and mortality from AKI.

Author (year)	Country	Sample size	Male sex (%)	Mean/median age (years)	ICU admission rate (%)	AKI (%)	AKI Stage 1 (%)	AKI Stage 2 (%)	AKI Stage 3 (%)	AKI patient deaths (%)
Sindhu et al. (2022)	India	2650	81.6%	62.6	4.4%	190 (72.0%)	71.0%	15.3%	13.7%	42 (22.1%)
Bell et al. (2021)	England	448	54.8%	69.4	13.8%	118 (26.3 %)	55.1%	18.6 %	26.3 %	64 (54.3%)
Scarpioni et al. (2021)	Italy	1701	64.3%	72.8	–	233 (13.7%)	65.0%	15.0%	17.0%	132 (56.7%)
Chen et al. (2021)	China	1851	48.0%	62.0	29%	115 (6.7%)	61.4%	22.8%	15.8%	37 (32.2%)
Sullivan et al. (2021)	UK	41294	62.6%	68.0	–	13000 (31.5%)	65.9%	20.1%	14.1%	5252 (40.4%)
Rahimzadeh et al. (2021)	Iran	516	62.8%	57.6	15.3%	194 (37.6%)	61.9%	18.0%	20.1%	77 (39.7%)
Hung et al. (2022)	African	990	92.1%	68.0	–	392 (39.6%)	64.0%	15.1%	20.9%	102 (26.0%)
Marques et al. (2021)	Portugal	544	56.3%	66.2	–	339 (62.3%))	32.2%	13.6%	54.3%	61 (18.0%)
Kilis‐pstrusinska et al. (2021)	Poland	1958	52.1%	62.3	11.5%	237 (12.1%)	–	–	–	146 (61.6%)
Strohbehn et al. (2021)	USA	1091	49.5%	67.0	–	251 (23.0%)	44.2%	25.9%	29.9%	81 (32.0%)
Hsu et al. (2022)	USA	4221	63.5%	61.0	–	2361 (56.0%)	22.3%	19.8%	57.9%	1458 (61.8%)
Jewell et al. (2021)	UK	1248	58.8%	69.0	18.2%	487 (39.0%)	51.0%	13.0%	36.0%	216 (44.4%)
Procaccini et al. (2021)	Spain	3182	–	72.0	–	548 (17.22%)	70.1%	19.3%	10.6%	211 (38.5%)
Wan et al. (2021)	UK	1855	60.5%	65.0	18.2%	455 (24.5%)	44.0%	19.8%	36.3%	242 (53.2%)

COVID-19, coronavirus disease 2019; AKI, acute kidney injury.

## SARS-CoV-2 Directly Invades Kidney Host Cells

COVID-19 is a respiratory infectious disease caused by SARS-CoV-2 infection. With more than 420 million confirmed COVID-19 cases worldwide since its onset in February 2020 (https://www.worldometers.info/coronavirus/). The lungs and immune system are the most common and most critical organs that are damaged but other organs, including the kidneys, heart, liver, digestive tract, and male reproductive system (He et al., 2020a), are also damaged to varying degrees (He et al., 2020b). Coronavirus has caused three pandemics in human history, including SARS-CoV-2, SARS-CoV, and the Middle East respiratory syndrome coronavirus (MERS-CoV), all of which belong to the β-coronavirus family. Also, the genome sequences of SARS-CoV-2 and SARS-CoV have nearly 80% similarity (Al-Qahtani, 2020). The spike proteins of these coronaviruses have a similar 3D structure which has a strong binding affinity to the cell receptor ACE2. After binding to ACE2, the spike protein of the virus is activated and cleaved by the cell TMPRSS2 and then the virus releases fusion peptides to enter cells (Shang et al., 2020). In addition, SARS-CoV-2 has its own furin cleavage sequence, which may enhance the affinity of the virus to host cells (Walls et al., 2020; Coutard et al., 2020). Previous studies showed that infection with SARS-CoV and MERS-CoV can cause kidney injury. Chu et al. reported that among 536 patients with SARS, 36 (6.7%) had AKI, and 33 (91.7%) of them eventually died. The autopsy report revealed that different degrees of acute tubular necrosis were observed under the microscope (Chu et al., 2005). The identification and isolation of SARS-CoV in renal epithelial Vero E6 cells also provides a reasonable explanation for the invasion of SARS-CoV into kidney host cells. Different from SARS, renal failure is the main manifestation of renal damage in MERS. (Al Ghamdi et al., 2016; Arabi et al., 2019). In a study of 30 patients with MERS-CoV infection, up to half of the patients had proteinuria, and 8 (26.7%) had AKI (Cha et al., 2015). Autopsy results showed that MERS-CoV particles were localized in the pneumocytes, pulmonary macrophages, and renal proximal tubular epithelial cells (Alsaad et al., 2018), indicating that viral kidney tropism is a potential AKI mechanism. The similarities in the genomes and invasion methods of SARS-CoV-2, SARS-CoV, and MERS-CoV provide the possibility for SARS-CoV-2 to invade kidney host cells directly.

**Figure 1:**
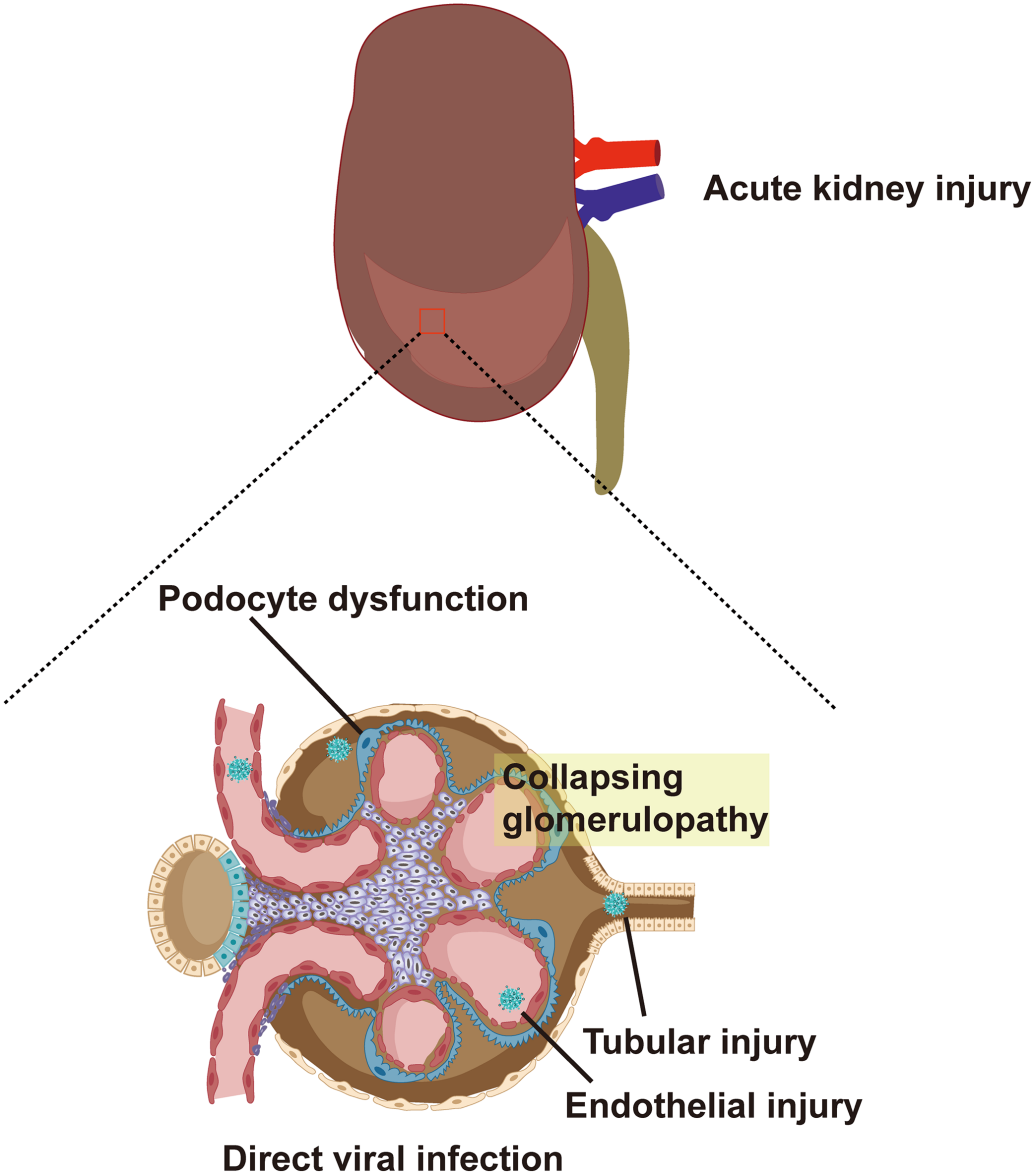
ACE2 and TMPRSS2 are expressed in proximal convoluted tubule cells, proximal straight tubule cells and podocytes. SARS-CoV-2 enters glomerular capillaries through the blood circulation, and then invades podocytes or enters renal tubular fluid to contact receptors in proximal tubules. This results in podocyte dysfunction, tubular injury, endothelial injury and collapsing glomerular disease. ACE2, angiotensin-converting enzyme 2; TMPRSS2, type II transmembrane serine protease; SARS-CoV-2, Severe Acute Respiratory Syndrome Coronavirus 2.

In terms of virus invasion mechanism, the expression of ACE2, TMPRSS2, and furin, which are required for SARS-CoV-2 to invade cells, has been detected in lung macrophages, kidney, and adrenal stromal cells strongly suggesting that these organs are susceptible to COVID-19 (Zhou et al., 2020a). A study further determined the expression levels of ACE2 and TMPRSS2 in the kidneys through the violin and scatter plots generated with reduction of UMAP, and revealed that ACE2 and TMPRSS2 intersected and were expressed in proximal convoluted tubule cells, proximal straight tubule cells, and podocytes (He et al., 2020b). It provides a theoretical basis for the potential entry pathway of SARS-CoV-2 to invade proximal renal tubular cells and podocytes. SARS-CoV-2 first infects the respiratory tract and it needs to be transported through the blood to reach the kidneys. According to reports, 10%-15% of patients with COVID-19, especially critically ill patients, suffer from SARS-CoV-2 RNAemia (Chen et al., 2020b; Huang et al., 2020). Moreover, Wichmann et al. reported autopsy results of 12 patients with COVID-19, six of whom had SARS-CoV-2 RNAemia and viral RNA was detected in their kidney tissue (Wichmann et al., 2020). Finally, the presence of SARS-CoV-2 in urine indicates that the renal tubules are directly exposed to the virus and may directly interact with the virus (Chu et al., 2005; Diao et al., 2021b). Therefore, the virus may enter the glomerular capillaries through the blood circulation and then invade podocytes or enter the renal tubule fluid to contact the receptors in the proximal tubules (**Figure 1**) (Ahmadian et al., 2021). Although the expression of ACE2 in the proximal tubules’ brush border apical membrane in the kidney is higher than that in the lung cells, the expression of TMPRSS2 is lower in the proximal tubule cells of the kidney (Ye et al., 2006). A recent study reported the discovery of a potential substitute for TMPRSS2 in proximal tubule cells. This study found that SARS-CoV-2 attacks target cells through the transmembrane glycoprotein CD147 (Su et al., 2020; Wang et al., 2020a). In addition, other proteases are expressed in kidney cells, including glutamyl aminopeptidase, cathepsin B/L, cysteine, and serine protease dipeptidyl peptidase 4 (DPP4). These proteases may promote SARS -CoV-2 and ACE2 binding and virus entry (Soleimani, 2020).

Clinical evidence also suggests that kidney involvement is common. In a large-scale prospective study, 701 patients with COVID-19 experienced increased serum creatinine, blood urea nitrogen, hematuria, and proteinuria (Cheng et al., 2020). However, the incidence of AKI varies greatly between different studies. Yang et al. reported that severe COVID-19 patients are more susceptible to AKI. In their study, 29% of severely ill patients with COVID-19 progressed to AKI (Yang et al., 2020). Data from over 5,000 patients with COVID-19 from a large medical network in the New York metropolitan area show that 37% of hospitalized patients had AKI and 14% of them required renal replacement therapy (Hirsch et al., 2020). Conversely, two large clinical studies indicate that the prevalence of AKI among hospitalized patients with COVID-19 is relatively low (0.5-5%) (Cheng et al., 2020; Guan et al., 2020). There is no doubt that AKI is a significant non-respiratory clinical manifestation of patients with COVID-19 in different clinical studies. According to the conclusion drawn from single-cell transcriptome analysis, the cytopathic effect of SARS-CoV-2 on podocytes and proximal tubule cells may be the cause of AKI (Pan et al., 2020). Farkash et al. found that the virus particles in the renal tubular epithelium are morphologically identical to SARS-CoV-2 (Farkash et al., 2020). In addition, Su et al. reported the detection of SARS-CoV-2 particles in renal tubular cells and podocytes, showing that the virus first invades podocytes and then enters the tubular fluid to bind to ACE2 in the proximal tubules to invade the renal parenchyma (Su et al., 2020). However, not all reports of COVID-19-related kidney pathological changes are consistent. Akilesh et al. performed a biopsy study of severely ill COVID-19 patients, which showed that although the patients had manifestations of acute tubular injury, immunohistochemistry for SARS-CoV-2 nucleocapsid and RNA *in situ* hybridization (ISH) for viral genomes of all four patient samples were negative, and direct viral invasion of the kidney could not be determined (Akilesh et al., 2021). Therefore, to determine whether SARS-CoV-2 directly invades kidney cells, cohort studies including kidney biopsy results from large numbers of COVID-19 patients are needed.

## Indirect Kidney Injury Caused by SARS-CoV-2 Infection

### Dysregulated Immune Responses and Cytokine Storm

The immune system is also a common and important organ affected by SARS-CoV-2 infection. During SARS-CoV-2 infection, neutrophils, leukocytes, and neutrophil-lymphocyte ratios were significantly increased in patients with severe COVID-19 compared with patients with mild COVID-19 (Qin et al., 2020). Kidney biopsy of patients with COVID-19 presents high levels of CD4+ T cells, CD56+ natural killer cells, and CD68+ macrophages infiltrated into the tubulo-interstitium. This condition indicates that T cells are activated and subsequently migrate to the site of infection to function. However, SARS-CoV-2 affects tissues under an ineffective immune response and promotes the necrosis or apoptosis of T cells by releasing cytokine storms, thereby reducing T cells and impairing virus clearance (Chen et al., 2020a; Huang et al., 2020). Decreased lymphocyte counts in COVID-19 patients are known to often lead to poor prognosis (Tan et al., 2020). According to clinical data, lymphopenia was found in 40% of patients with COVID-19 (Zheng et al., 2020). Liu et al. observed an inverse correlation between T-cell counts and kinetic changes in cytokine levels in patients with severe COVID-19 (Liu et al., 2020). After 4-6 days of onset, the serum interleukin-10 (IL-10), IL-2, IL-4, tumor necrosis factor-alpha (TNF-α), and interferon-gamma (IFN-γ) levels increased significantly along with the decline in T cell counts. While the number of T cells was restored, the levels of IL-6, IL-10, IL-2, IL-4, TNF-α and IFN-γ in serum decreased (Liu et al., 2020). In order to understand the dynamics of the immune response in COVID-19 patients and its association with clinical outcomes, Lucas et al. analyzed peripheral blood mononuclear cell and plasma samples from patients with moderate or severe COVID-19 and healthy donors by flow cytometry and enzyme linked immunosorbent assay (Lucas et al., 2020). They observed a “key COVID-19 feature” shared by the moderate and severe disease groups, which they defined as the following inflammatory cytokines: IL-1α, IL-1β, IL-17A, IL-12 p70, and IFNα. And this study found that after day 10 of infection, these markers began to decline in patients with moderate disease, while levels of these key markers remained elevated in patients with severe COVID-19 (Lucas et al., 2020). In addition, Lucas et al. reported early cytokines that may predict disease outcome, including eotaxin 3, IL-33, Thymic Stromal Lymphopoietin, IL-21, IL-23, IL-17F, IFN-γ, IL- 12 p70, IL-15, IL-2, TNF, IL-4, IL-5, IL-13, IL-1α, IL-1β, IL-17A, IL-17E, IL-22 and many chemokines involved in leukocyte trafficking, these markers are associated with coagulation dysfunction and higher mortality in COVID-19 patients (Lucas et al., 2020).

Recently, the presence of autoantibodies to ACE2 was confirmed in the sera of individuals with severe COVID-19, leading to the emergence of the doctrine of autoimmunity in COVID-19 patients (Casciola-Rosen et al., 2020). Binding of soluble ACE2 to SARS-CoV-2 underlies ACE2 autoimmunity. Soluble ACE2, also known as serum or plasma ACE2, is commonly found in the serum of patients with hypertension and heart disease (Epelman et al., 2008). McMillan et al. observed that a complex of SARS-CoV-2 and soluble ACE2 forms and enters the blood circulation of infected patients (McMillan et al., 2021). Binding of ACE2 to the SARS-CoV-2 spike protein induces a conformational change in both proteins, providing a target for the formation of autoantibodies (Zhang et al., 2005) resulting in the production of antibodies against ACE2. Antibodies trigger type 2 and type 3 hypersensitivity reactions, as well as type 4 hypersensitivity reactions after complexes of SARS-CoV-2 and soluble ACE2 are processed by antigen-presenting cells. During SARS-CoV-2 infection, triggering of type 2 hypersensitivity reactions produces immunoglobulin M (IgM) against and ACE2, which targets ACE2 in kidney cells, resulting in renal impairment. Multiple studies of renal biopsies from patients with COVID-19 have confirmed the impact of autoimmunity on renal function. Winkler et al. observed IgG, IgM and C3 deposition in the glomerular basement membrane of patients with COVID-19 (Winkler et al., 2021). Macor et al. found IgG and C deposits around the tubules and glomeruli after detailed analysis of kidney sections from patients with COVID-19 (Macor et al., 2021). In addition, a study found IgA granular deposition in the renal mesangium of patients with COVID-19 by immunofluorescence and the disappearance of podocytes under electron microscopy, and the study proposed that these pathological changes were associated with type 3 hypersensitivity reactions triggered by antigen-antibody complexes (Jedlowski and Jedlowski, 2022). Since ACE2 is widely expressed in different organs of the human body, it is very necessary to further study the role of anti-ACE2 autoantibodies in the pathogenesis of COVID-19. In particular, anti-ACE2 autoantibodies may unbalance the ratio of ACE to ACE2, leading to renin-angiotensin system (RAS) disorders, promoting tissue damage and worsening inflammation. The pathogenesis of AKI in COVID-19 also involves the complement system (Noris et al., 2020). Earlier studies have shown that after accumulating in the lumen of the renal tubules, complement C5b-9 accumulates at the brush borders of the apical tubules through an alternative pathway, leading to tubulointerstitial damage (David et al., 1997; Cybulsky et al., 2002). One study performed renal biopsies from six severely ill COVID-19 patients and observed extensive complement deposition on renal tubules, suggesting that SARS-CoV-2 infection can activate complement deposition and play a role in renal injury (Noris et al., 2020).

**Figure 2:**
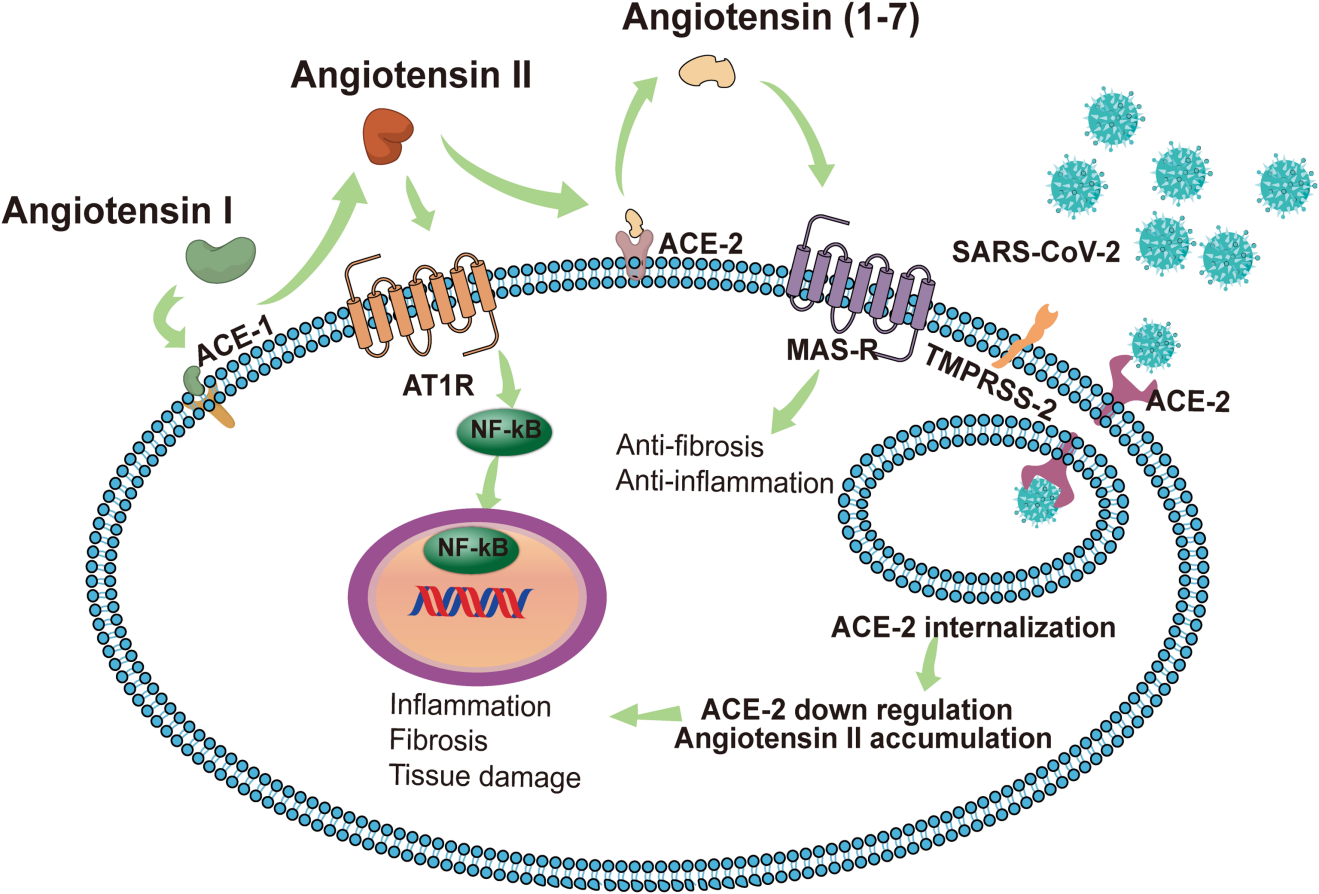
In normal conditions with a balanced RAS, renin cleaves angiotensinogen to produce Ang I, and then Ang I is cleaved by ACE1 to Ang II. The combination of Ang II and AT1R activates the NF-κB signaling pathway, modulates the gene expression of inflammatory cytokines, and induces harmful effects such as fibrosis, inflammation and tissue damage. ACE2 induces the cleavage of Ang II into Ang 1-7. Subsequently, Ang 1-7 activates MAS-R and induces anti-inflammatory and anti-fibrosis effects. SARS-CoV-2 identifies ACE2 as a receptor to invade host cells. The endocytosis of the virus reduces the expression of ACE2 on the cell membrane, thereby impairing the protective effect of the ACE2/Ang 1-7/MAS-R axis. Accumulated Ang II leads to the excessive activation of AT1R, induces the release of a variety of inflammatory cytokines, and even causes a cytokine storm. SARS-CoV-2 can also invade immune cells (CD4+ T cells and CD8+ T cells) by binding with ACE2, causing immune response dysregulation. The intensified cytokine storm and immune response dysregulation may have indirect impacts on multi‐organ failure, especially the kidneys. AKI, acute kidney injury; SARS-CoV-2, Severe Acute Respiratory Syndrome Coronavirus 2; ACE2, angiotensin-converting enzyme 2; RAS, renin-angiotensin system; Ang II, angiotensin II; AT1R, angiotensin receptor type 1; MAS-R, MAS receptor.

In some cases, persistent influence of viral antigens, high levels of pro-inflammatory factors, and cellular damage-associated molecular patterns (DAMPS) can exacerbate an immune response that progresses from a localized immune response to a systemic inflammatory response known as “cytokine storm”. which subsequently causes systemic inflammatory response syndrome, leading to multi-organ dysfunction (Tay et al., 2020). Karki et al. described links between the cytokine storm and the programmed cell death (PCD) process, a PCD activated by a virus (such as influenza A virus) and triggered by cytokines (Karki and Kanneganti, 2021). They pointed out that cytokines are intricately related to cell death mechanisms and are involved in a positive feedback loop. Among the numerous pro-inflammatory cytokines that are elevated during the cytokine storm, IL-1, IL-6, TNF, and IFN-γ are crucial. Of these, TNF and IFN-γ have been extensively studied and they independently induce apoptosis or necroptosis (Karki and Kanneganti, 2021). The up-regulated pro-inflammatory genes in patients with severe COVID-19 are mainly located in the NF-κB and type I IFN signaling pathways. *In vitro* experiments have shown that healthy peripheral blood mononuclear cells infected with SARS-CoV-2 have increased pro-inflammatory cytokines, including TNF, IL-6, IFN-γ and IL-1β. Of these, TNF and IFN-γ are highly upregulated in the serum of patients with severe COVID-19 and TNF- and IFN-γ-induced cell death can lead to systemic inflammation, tissue damage, multiple organ failure, and death of COVID-19 patients (Karki and Kanneganti, 2021). In addition, elevated cytokines mediate inflammatory cells that adhere to the endothelial cells of the kidney, which can cause kidney damage (He et al., 2005). The formation of a cytokine storm is related to the imbalance of the RAS (**Figure 2**). As a key receptor recognized by SARS-CoV-2 and a key enzyme in the renin-angiotensin system (Hoffmann et al., 2020), ACE2 inactivates angiotensin II (Ang II) to Ang (1-7) and converts angiotensin I (Ang I) to Ang (1-9). Conversely, ACE inactivates Ang I to Ang II and converts Ang (1-9) to Ang (1-7) (Vickers et al., 2002). Ang II plays an important role in RAS by acting on the angiotensin type 1 receptors (AT1R) and AT2R. Of the two, AT1R is activated by Ang II to regulate aldosterone release in the adrenal cortex and plays a key role in fluid balance. In addition to this, AT1R activation can promote thrombosis, inflammation, and fibrosis (Karnik et al., 2015). By contrast, Ang (1-7) exerts anti-inflammatory effects through Mas receptor (MAS-R) and G proteins (Dilauro et al., 2010).

Once ACE2 is occupied by SARS-CoV-2, free Ang II will accumulate due to the lack of degradation by ACE2. This phenomenon may lead to the activation of AT1R and the reduction of angiotensin production (1-7), thereby triggering a cytokine storm (Iwasaki et al., 2021). Furthermore, Ang II interacts with kidney resident cells to promote the production of pro-inflammatory factors, including prostaglandins, vascular endial cell growth factor, nuclear factor kappa B, TNFα, IL-1β, IL-6, and IFN-γ (Williams et al., 1995; Fyhrquist and Saijonmaa, 2008). It also stimulates the production of cytokines/chemokines to cause immune cells (neutrophils, mononuclear cells, T cells, and B cells) to infiltrate the injury site and enhance the inflammatory response (Nataraj et al., 1999). These factors contribute to the AKI in COVID-19 cases by promoting tubular and endothelial dysfunction. Dealing with RAS imbalances will be the key to the problem. Currently, the conventional drugs acting on the RAS are ACE inhibitors (ACEI) and angiotensin receptor blockers (ARB). ACEI and ARB block the various biological effects of Ang II by reducing the production of Ang II and blocking the activation of AT1R, respectively, thereby reducing the risk of inflammation and thrombosis. However, *In vitro* and animal studies have shown that the use of ACEI and ARB causes an increase in the expression and activity levels of ACE1 and ACE2 (Ferrario et al., 2005). The increased expression of ACE2 facilitates the recognition of target receptors by SARS-CoV-2 to invade cells and adversely affects the progression of COVID-19. Tetlow et al. analyzed data from COVID-19 patients at an inner London Hospital to determine whether ACEI or ARB use was associated with AKI, incidents of thrombosis, and in-hospital mortality. Results showed no association between ACEI/ARB use and in-hospital mortality in patients, and there was no evidence that the use of these drugs led to the development of AKI or the formation of microvascular thrombosis (Tetlow et al., 2021). Conversely, due to the anti-inflammatory effects of Ang (1-7), increased ACE2 expression might also induce beneficial effects in COVID-19 (Dilauro et al., 2010). While the impact of RAS inhibitor-induced ACE2 upregulation on clinical outcomes in COVID-19 remains unclear, there has emerged an overwhelming consensus that despite possible ACE2 upregulation, the use of ACEIs and ARBs did not lead to poor prognostic outcomes (Cook and Ausiello, 2021).

### Organ Crosstalk

**Figure 3:**
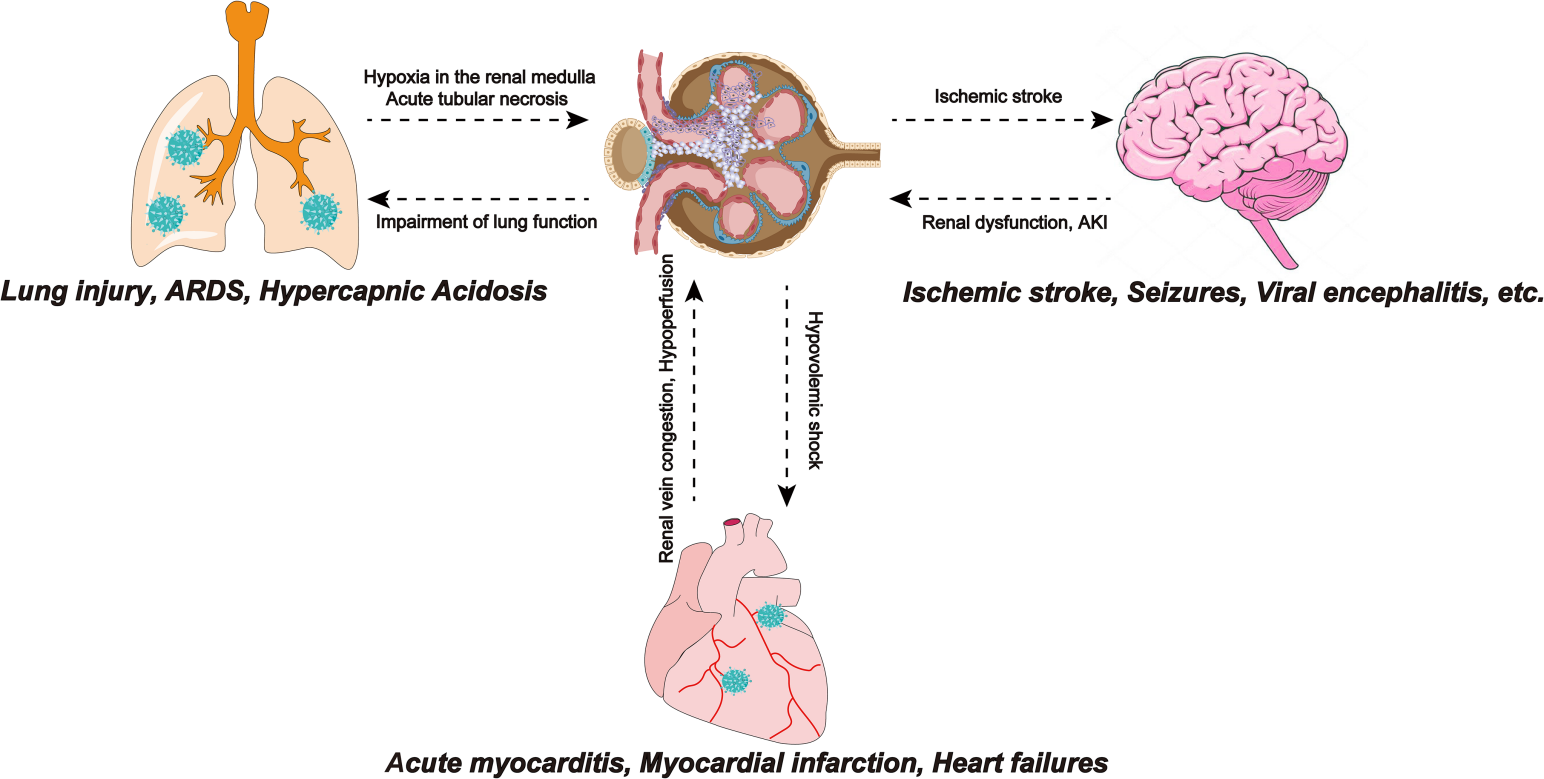
SARS-COV-2 infection leads to ARDS, and insufficiency of lung function leads to hypoxia of the renal medulla, causing acute tubular necrosis. Also, impaired lung function can lead to hypercapnic acidosis, which leads to renal failure by reducing GFR. Myocarditis caused by SARS-CoV-2 infection reduces cardiac output, causing renal congestion and further impairing its perfusion. The renal response to myocardial dysfunction may lead to hypovolemic shock, which can adversely affect cardiorespiratory function. Ischemic stroke events in COVID-19 patients are not uncommon and have been associated with the hypercoagulable state effect caused by SARS-CoV-2. Focal cerebral ischemia leads to sympathetic hyperactivity, which leads to the progression of kidney damage. SARS-COV-2, Severe Acute Respiratory Syndrome Coronavirus 2; ARDS, Acute respiratory distress syndrome; GFR, glomerular filtration rate; SARS-CoV-2, Severe Acute Respiratory Syndrome Coronavirus 2; COVID-19, coronavirus disease 2019.

Acute respiratory distress syndrome (ARDS) is one of the serious complications of COVID-19. ARDS in COVID-19 patients involves two pathological mechanisms, first, as a receptor recognized by SARS-COV-2, ACE2 is located in pneumocyte type II (Tian et al., 2020). Therefore, direct virus invasion destroys alveolar cells and reduces pulmonary surfactant, leading to ARDS. The second mechanism is a cytokine storm, in which the SARS-COV-2 infection produces large amounts of pro-inflammatory cytokines and an excessive inflammatory response, leading to multiple organ failures, including the kidneys and ARDS (Elrobaa and New, 2021). Crosstalk between the lungs and kidneys was observed in ARDS (Panitchote et al., 2019). A retrospective study showed that of 375 ARDS patients without a history of CKD and/or AKI, approximately 70% developed AKI (Panitchote et al., 2019). The reason may be that ARDS leads to hypoxia in the renal medulla, which consequently leads to acute tubular necrosis. In addition, the impairment of lung function caused by COVID-19 can lead to hypercapnic acidosis. Bratel et al. pointed out that hypercapnia due to pulmonary dysfunction leads to renal failure by alleviating glomerular filtration rate (GFR) (Bratel et al., 2003). Finally, ARDS patients require a ventilator for breathing and the artificial positive pressure generated by the ventilator affects intrathoracic pressure, which reduces cardiac output and reduces GFR, thereby affecting renal function. The lungs and kidneys work together to maintain electrolyte balance and acid-base balance in the body. Therefore, impairment of renal function will disturb the balance and also affect lung function. In addition to lung function, the kidneys also have crosstalk with the heart and brain (**Figure 3**).

The crosstalk between the heart and the kidneys may also be related to AKI in patients with COVID-19 (Ronco and Reis, 2020). In the early stages of COVID-19, cardiovascular system dysfunctions such as acute myocarditis, myocardial infarction, and heart failure often occur. Myocarditis caused by SARS-CoV-2 infection reduces cardiac output and end-organ perfusion and the accompanying right ventricular dysfunction can lead to venous congestion, causing kidney congestion and further impairing its perfusion. Renal vein congestion, hypotension, and subsequent hypoperfusion decrease the glomerular filtration rate (Ronco and Reis, 2020). In addition, the response of the kidneys to myocardial dysfunction may cause hypovolemic shock, which can exert detrimental effects on heart and lung functions (Scrascia et al., 2017; Gautier-Vargas et al., 2020).

Neurological manifestations have also been reported recently in patients with COVID-19. Mild cases are characterized by headache, dizziness, smell, and taste dysfunction and severe cases are accompanied by ischemic stroke, seizures, motor, and sensory deficits (Liotta et al., 2020). Several studies have reported viral encephalitis in patients with COVID-19 and found SARS-CoV-2 in their cerebrospinal fluid (Wang et al., 2020b). It is known that ACE2 is expressed in the human brain (Hamming et al., 2004). The mechanism of the nervous system manifestations of COVID-19 may be that SARS-CoV-2-related cytokines such as IL-1b, IL-17, IL-6, and TNF alter the permeability of the blood-brain barrier, which makes SARS-CoV-2 can reach the brain and recognize ACE2 directly affects brain cells (Iadecola et al., 2020). The hypercoagulable state caused by SARS-CoV-2 infection can lead to ischemic stroke in patients with COVID-19 (Khismatullin et al., 2021). Accumulating studies have observed an association between ischemic stroke and renal dysfunction. Cai et al.’s study of ischemic stroke patients showed that cerebral cortical infarction is an independent risk factor for AKI. Focal cerebral ischemia leads to sympathetic hyperactivity that contributes to the progression of renal injury (Cai et al., 2021).

### Endothelial Dysfunction and Hypercoagulation and Other Mechanisms

The expression of ACE2 in vascular endothelial cells provides a pathophysiological basis for virus invasion. Emerging evidence also suggests that endothelial dysfunction plays a contributing role in the development of renal dysfunction in patients with COVID-19. Severe COVID-19 cases are frequently characterized by microvascular damage (Tang et al., 2020; Zhou et al., 2020b). The histopathology of three patients with COVID-19 revealed that SARS-CoV-2 directly infects endothelial cells, causing diffuse endothelial inflammation (Varga et al., 2020). Therefore, microvascular inflammation and dysfunction are likely to cause multiple organ failure (including kidneys) in patients with COVID-19.

Furthermore, activation of complement cascades and a hypercoagulable state have a potential impact on the development of AKI in patients with COVID‐19 (Taverna et al., 2021). The increased coagulation activity of most patients with severe COVID-19 leads to microvascular thrombosis (Terpos et al., 2020). From the onset of cytokine storm, the activation of DAMPS and coagulation factors promote the hypercoagulable state (Delvaeye and Conway, 2009). The endothelial damage caused by the virus may also be exposed to tissue factor, thereby stimulating the extrinsic coagulatory pathway. In addition, damaged endothelial cells recruit neutrophils, release neutrophil extracellular traps, and stimulate the coagulation contact pathway by activating platelets (Merad and Martin, 2020). Finally, hypoxia caused by COVID-19 can lead to thrombosis (Khismatullin et al., 2021). In a hypercoagulable state, acute tubular necrosis may progress to cortical necrosis and cause irreversible kidney injury. The state of microthrombosis and microangiopathy can also increase the risk of microinfarction in other organs, leading to multiple tissue damage.

Sepsis is also an indirect cause of AKI in COVID-19 patients. It is caused by the cytokine storm cascade after viral infection (Jin et al., 2020). A retrospective study, conducted in Wuhan, China, showed that the incidence of sepsis among 191 COVID-19 patients was 59% (Zhou et al., 2020b). Another study revealed that 6.4% of 113 severe COVID-19 patients had septic shock and these patients may develop septic AKI and trigger kidney damage (Jin et al., 2020; Ronco and Reis, 2020).

An increasing number of studies are starting to note the effects of drugs applied during COVID-19 treatment on kidney function. The use of nephrotoxic drugs, such as vancomycin, is also a potential factor in AKI (Na et al., 2020). Fontana et al. pointed out that the side effects caused by vitamin C application during COVID-19 treatment may be underestimated (Fontana et al., 2020). Fontana et al. performed renal biopsies in two patients with renal insufficiency with COVID-19 and found diffuse calcium oxalate monohydrate crystals in the renal tubules of these two patients, which led to tubular damage. The authors then diagnosed these two patients with oxalate nephropathy associated with vitamin C use (Fontana et al., 2020). Previous studies demonstrated that high doses of vitamin C significantly reduced proinflammatory cytokines, C-reactive protein, and procalcitonin in patients with sepsis (Fowler et al., 2014). The potential inhibitory role of vitamin C in the cytokine storm induced by SARS-Cov-2 infection has received increasing attention. Unfortunately, vitamin C causes hyperoxaluria through endogenous conversion of ascorbic acid to oxalate (Cossey et al., 2013). Under normal physiological conditions, crystals pass rapidly through the renal tubules. However, renal tubular damage caused by COVID-19 leads to retention of crystals in the tubes and may induce calcium oxalate calculi. In addition to the effects of vitamin C, some antibiotics, such as ceftriaxone, can form crystals in the urine (Daudon et al., 2017). The altered gut microbial milieu and overabsorption of oxalate caused by COVID-19-associated enteritis can also cause hyperoxaluria (Wan et al., 2020). Therefore, for COVID-19 patients with renal insufficiency, medication should be used with caution according to the specific situation.

**Figure 4:**
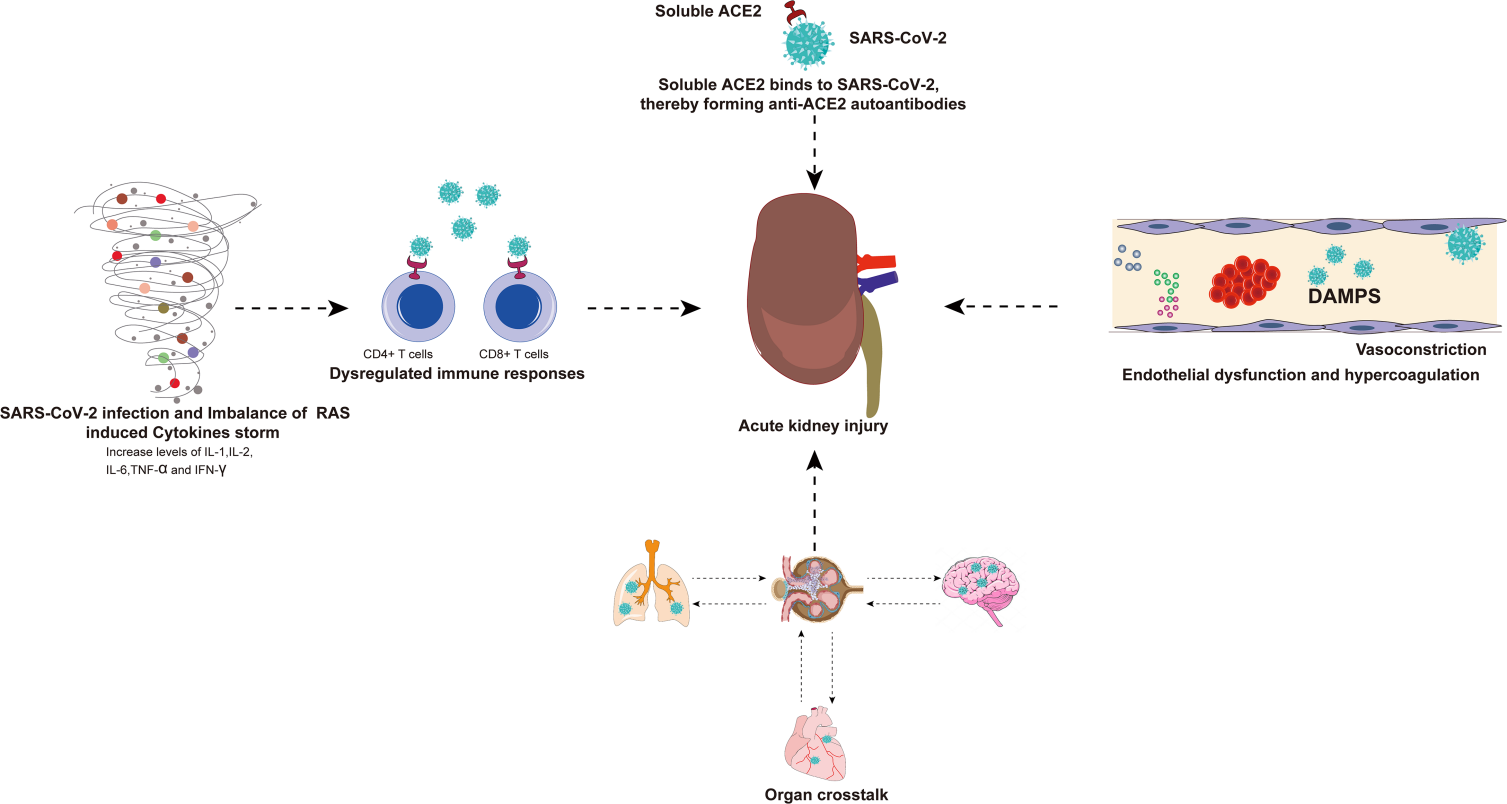
There are many indirect factors in AKI caused by SARS-CoV-2 infection. Viral infection and RAS imbalance induce the release of various inflammatory cytokines and even trigger cytokine storm. SARS-CoV-2 can also invade immune cells (CD4+ T cells and CD8+ T cells) by binding to ACE2, resulting in a dysregulated immune response. The exacerbation of cytokine storms and dysregulated immune responses may have indirect effects on multiple organ failure, especially the kidneys. The virus-infected lungs, heart, and brain will have organ crosstalk with the kidneys, which will further aggravate kidney damage. Binding of SARS-CoV-2 and soluble ACE2 induces a conformational change in both proteins, providing a target for the formation of autoantibodies that generate antibodies against ACE2, resulting in kidney damage. In addition, endothelial dysfunction, complement dysregulation, and hypercoagulability have also been associated with AKI caused by SARS-CoV-2 infection. AKI, acute kidney injury; SARS-CoV-2, Severe Acute Respiratory Syndrome Coronavirus 2; RAS, renin-angiotensin system; ACE2, angiotensin-converting enzyme 2.

There are many indirect factors in AKI caused by SARS-CoV-2 infection and these factors are summarized in the figure for ease of understanding (**Figure 4**).

## Possible Treatment and Management of Patients With AKI During SARS-CoV-2 Infection

Throughout the COVID-19 pandemic, the onset of AKI portends a poor prognosis. Wang et al. observed that remdesivir, previously used against SARS-CoV and MERS-CoV, had no apparent effect on clinical outcomes in COVID-19 (Wang et al., 2020c). Non-invasive ventilation, high-flow nasal cannula, and corticosteroids are the main methods of support. Currently, many treatments and management measures for COVID-AKI are in clinical trials. ACEI and ARB treat many cardiovascular and renal diseases by reducing AT1R activation and upregulating ACE2 expression (Carey, 2015). However, there are concerns about whether upregulation of ACE2 increases the chance of SARS-CoV-2 to recognize its invading cells. In fact, several studies have reported that the use of RAS inhibitors is associated with a lower risk of infection and mortality in COVID-19 patients (Baral et al., 2021). Rahmani et al. reported that the application of losartan did not upregulate total ACE2 levels in human renal tubular cells. Interestingly, Rahmani et al. found that losartan upregulated interferon-stimulated genes in podocytes and renal tubular cells to limit SARS-CoV-2 infection. The authors show that losartan prevents ACE2 internalization and mitigates SARS-CoV-2 infection in renal tubular cells. Therefore, losartan represents a potential adjunctive therapy (Rahmani et al., 2022). In severe cases, “cytokine storm” can be observed in the serum of COVID-19 patients. A variety of treatments, including antibody therapy and plasma exchange, can directly remove cytokines and relieve “cytokine storm” (Swol and Lorusso, 2020). In addition, plasma exchange therapy can relieve sepsis caused by viral superimposed bacterial infection. As previously mentioned, both the hyperinflammatory state and sepsis are indirect contributors to AKI. Therefore, therapeutic plasma exchange can be one of the treatment options in the case of severe disease.

## Conclusion

COVID-19 has now constituted a global pandemic and the number of confirmed cases is still increasing. As a common comorbidity of COVID-19, AKI is an indicator of negative prognosis and disease severity (Nadim et al., 2020; Richardson et al., 2020; Varga et al., 2020). AKI caused by SARS-CoV-2 infection results from both direct and indirect injury. Direct injury is caused by the SARS-CoV-2 virus targeting and infecting kidney cells (such as proximal tubule cells and podocytes expressing ACE2 and TMPRSS2). Indirect injury mainly results from an immune response disorder, a cytokine storm, endothelial injury, and organ crosstalk. COVID-19‐induced AKI is associated with high mortality in hospitalized patients (Soleimani, 2020) and there is currently no specific anti-SARS-CoV-2 treatment. Therefore, understanding the pathogenesis of AKI in COVID-19 patients is of great significance for improving the prognosis of COVID-19 patients. Keeping in mind the risk of AKI for COVID-19 patients and reducing renal tubular damage will benefit COVID-19 patients. In addition, further research is needed to improve the understanding of AKI secondary to COVID-19 in order to obtain sufficient evidence to develop new preventive measures and treatments.

## Author Contributions

WH, XL and BH searched the literature and conceived and wrote the review. DL, LC, YL, JD, YT and SX revised the paper, tables and graphic abstract. GW and BF critically appraised the literature and made an intellectual contribution to the work. All authors read and approved the final manuscript.

## Funding

The present study was supported by the National Natural Science Foundation of China (Grant nos. 81560419 and 81960512).

## Conflict of Interest

The authors declare that the research was conducted in the absence of any commercial or financial relationships that could be construed as a potential conflict of interest.

## Publisher’s Note

All claims expressed in this article are solely those of the authors and do not necessarily represent those of their affiliated organizations, or those of the publisher, the editors and the reviewers. Any product that may be evaluated in this article, or claim that may be made by its manufacturer, is not guaranteed or endorsed by the publisher.

## References

[B1] Ahmadian E. Hosseiniyan Khatibi S. M. Razi Soofiyani S. Abediazar S. Shoja M. M. Ardalan M.. (2021). Covid-19 and Kidney Injury: Pathophysiology and Molecular Mechanisms. Rev. Med. Virol. 31, 1–13. doi: 10.1002/rmv.2176 PMC7646060 33022818

[B2] Akilesh S. Nast C. C. Yamashita M. Henriksen K. Charu V. Troxell M. L.. (2021). Multicenter Clinicopathologic Correlation of Kidney Biopsies Performed in COVID-19 Patients Presenting With Acute Kidney Injury or Proteinuria. Am. J. Kidney Dis. 77, 82–93.e1. doi: 10.1053/j.ajkd.2020.10.001 33045255 PMC7546949

[B3] Al Ghamdi M. Alghamdi K. M. Ghandoora Y. Alzahrani A. Salah F. Alsulami A.. (2016). Treatment Outcomes for Patients With Middle Eastern Respiratory Syndrome Coronavirus (MERS CoV) Infection at a Coronavirus Referral Center in the Kingdom of Saudi Arabia. BMC Infect. Dis. 16, 1–7. doi: 10.1186/s12879-016-1492-4 27097824 PMC4839124

[B4] Al-Qahtani A. A. (2020). Severe Acute Respiratory Syndrome Coronavirus 2 (SARS-CoV-2): Emergence, History, Basic and Clinical Aspects. Saudi J. Biol. Sci. 27(10), 2531–2538. doi: 10.1016/j.sjbs.2020.04.033 32336927 PMC7179492

[B5] Alsaad K. O. Hajeer A. H. Al Balwi M. Al Moaiqel M. Al Oudah N. Al Ajlan A.. (2018). Histopathology of Middle East Respiratory Syndrome Coronovirus (MERS-CoV) Infection – Clinicopathological and Ultrastructural Study. Histopathology 72, 516–524. doi: 10.1111/his.13379 28858401 PMC7165512

[B6] Arabi Y. M. Deeb A. M. Al-Hameed F. Mandourah Y. Almekhlafi G. A. Sindi A. A.. (2019). Macrolides in Critically Ill Patients With Middle East Respiratory Syndrome. Int. J. Infect. Dis. 81, 184–190. doi: 10.1016/j.ijid.2019.01.041 30690213 PMC7110878

[B7] Baral R. Tsampasian V. Debski M. Moran B. Garg P. Clark A.. (2021). Association Between Renin-Angiotensin-Aldosterone System Inhibitors and Clinical Outcomes in Patients With COVID-19: A Systematic Review and Meta-Analysis. JAMA Netw. Open 4, e213594. doi: 10.1001/jamanetworkopen.2021.3594 33787911 PMC8013817

[B8] Bell J. S. James B. D. Al-Chalabi S. Sykes L. Kalra P. A. Green D. (2021). Community- Versus Hospital-Acquired Acute Kidney Injury in Hospitalised COVID-19 Patients. BMC Nephrol. 22, 1–11. doi: 10.1186/s12882-021-02471-2 34301204 PMC8299737

[B9] Bratel T. Ljungman S. Runold M. Stenvinkel P. (2003). Renal Function in Hypoxaemic Chronic Obstructive Pulmonary Disease: Effects of Long-Term Oxygen Treatment. Respir. Med. 97, 308–316. doi: 10.1053/rmed.2002.1401 12693791

[B10] Cai Y. Lu X. Cheng X. Lv Q. Xu G. Liu X. (2021). Increased Renal Dysfunction, Apoptosis, and Fibrogenesis Through Sympathetic Hyperactivity After Focal Cerebral Infarction. Transl. Stroke Res. doi: 10.1007/s12975-021-00900-w 33713029

[B11] Carey R. M. (2015). The Intrarenal Renin-Angiotensin System in Hypertension. Adv. Chronic Kidney Dis. 22, 204–210. doi: 10.1053/j.ackd.2014.11.004 25908469

[B12] Casciola-Rosen L. Thiemann D. R. Andrade F. Zambrano M. Rosen A. (2020). IgM Autoantibodies Recognizing ACE2 are Associated With Severe COVID-19. medRxiv. doi: 10.1101/2020.10.13.20211664

[B13] Cha R. H. Joh J. S. Jeong I. Lee J. Y. Shin H. S. Kim G.. (2015). Renal Complications and Their Prognosis in Korean Patients With Middle East Respiratory Syndrome-Coronavirus From the Central MERS-CoV Designated Hospital. J. Korean Med. Sci. 30, 1807–1814. doi: 10.3346/jkms.2015.30.12.1807 26713056 PMC4689825

[B14] Cheng Y. Luo R. Wang K. Zhang M. Wang Z. Dong L.. (2020). Kidney Disease is Associated With in-Hospital Death of Patients With COVID-19. Kidney Int. 97, 829–838. doi: 10.1016/j.kint.2020.03.005 32247631 PMC7110296

[B15] Chen K. Lei Y. He Y. Xiao F. Yu Y. Lai X.. (2021). Clinical Outcomes of Hospitalized COVID-19 Patients With Renal Injury: A Multi-Hospital Observational Study From Wuhan. Sci. Rep. 11, 1–12. doi: 10.1038/s41598-021-94570-1 34312430 PMC8313555

[B16] Chen D. Li X. Song Q. Hu C. Dai J. (2020a). Hypokalemia and Clinical Implications in Patients With Coronavirus Disease 2019 (COVID-19). Cold Spring Harbor Laboratory Press. doi: 10.1101/2020.02.27.20028530

[B17] Chen X. Zhao B. Qu Y. Chen Y. Xiong J. Feng Y.. (2020b). Detectable Serum Severe Acute Respiratory Syndrome Coronavirus 2 Viral Load (RNAemia) Is Closely Correlated With Drastically Elevated Interleukin 6 Level in Critically Ill Patients With Coronavirus Disease 2019. Clin. Infect. Dis. 71, 1937–1942. doi: 10.1093/cid/ciaa449 32301997 PMC7184354

[B18] Chu K. H. Tsang W. K. Tang C. S. Lam M. F. Lai F. M. To K. F.. (2005). Acute Renal Impairment in Coronavirus-Associated Severe Acute Respiratory Syndrome. Kidney Int. 67, 698–705. doi: 10.1111/j.1523-1755.2005.67130.x 15673319 PMC7112337

[B19] Cook J. R. Ausiello J. (2021). Functional ACE2 Deficiency Leading to Angiotensin Imbalance in the Pathophysiology of COVID-19. Rev. Endocr. Metab. Disord. 23,151–170. doi: 10.1007/s11154-021-09663-z 34195965 PMC8245275

[B20] Cossey L. N. Rahim F. Larsen C. P. (2013). Oxalate Nephropathy and Intravenous Vitamin C. Am. J. Kidney Dis. 61, 1032–1035. doi: 10.1053/j.ajkd.2013.01.025 23548555

[B21] Coutard B. Valle C. de Lamballerie X. Canard B. Seidah N. G. Decroly E. (2020). The Spike Glycoprotein of the New Coronavirus 2019-Ncov Contains a Furin-Like Cleavage Site Absent in CoV of the Same Clade. Antiviral Res. 176, 1–5. doi: 10.1016/j.antiviral.2020.104742 PMC7114094 32057769

[B22] Cybulsky A. V. Takano T. Papillon J. Khadir A. Liu J. Peng H. (2002). Complement C5b-9 Membrane Attack Complex Increases Expression of Endoplasmic Reticulum Stress Proteins in Glomerular Epithelial Cells. J. Biol. Chem. 277, 41342–41351. doi: 10.1074/jbc.M204694200 12191998

[B23] Daudon M. Frochot V. Bazin D. Jungers P. (2017). Drug-Induced Kidney Stones and Crystalline Nephropathy: Pathophysiology, Prevention and Treatment. Drugs 78, 1–39. doi: 10.1007/s40265-017-0853-7 29264783

[B24] David S. Biancone L. Caserta C. Bussolati B. Cambi V. Camussi G. (1997). Alternative Pathway Complement Activation Induces Proinflammatory Activity in Human Proximal Tubular Epithelial Cells. Nephrol. Dial. Transplant. 12, 51–56. doi: 10.1093/ndt/12.1.51 9027773

[B25] Delvaeye M. Conway E. M. (2009). Coagulation and Innate Immune Responses: Can We View Them Separately? Blood 114, 2367–2374. doi: 10.1182/blood-2009-05-199208 19584396

[B26] Diao B. Wang C. Wang R. Feng Z. Zhang J. Yang H.. (2021a). Human Kidney is a Target for Novel Severe Acute Respiratory Syndrome Coronavirus 2 Infection. Nat. Commun. 12, 1–9. doi: 10.1038/s41467-021-22781-1 33947851 PMC8096808

[B27] Diao B. Wen K. Zhang J. Chen J. Han C. Chen Y.. (2021b). Accuracy of a Nucleocapsid Protein Antigen Rapid Test in the Diagnosis of SARS-CoV-2 Infection. Clin. Microbiol. Infect. 27, 289.e1–289.e4. doi: 10.1016/j.cmi.2020.09.057 PMC7534827 33031947

[B28] Dilauro M. Zimpelmann J. Robertson S. J. Genest D. Burns K. D. (2010). Effect of ACE2 and Angiotensin-(1-7) in a Mouse Model of Early Chronic Kidney Disease. Am. J. Physiol. - Ren. Physiol. 298, 1523–1532. doi: 10.1152/ajprenal.00426.2009 20357030

[B29] Elrobaa I. H. New K. J. (2021). COVID-19: Pulmonary and Extra Pulmonary Manifestations. Front. Public Heal. 9. doi: 10.3389/fpubh.2021.711616 PMC8505777 34650947

[B30] Epelman S. Tang W. H. Chen S. Y. Van Lente F. Francis G. S. Sen S. (2008). Detection of Soluble Angiotensin-Converting Enzyme 2 in Heart Failure: Insights Into the Endogenous Counter-Regulatory Pathway of the Renin-Angiotensin-Aldosterone System. J. Am. Coll. Cardiol. 52, 750–754. doi: 10.1016/j.jacc.2008.02.088 18718423 PMC2856943

[B31] Farkash E. A. Wilson A. M. Jentzen J. M. (2020). Ultrastructural Evidence for Direct Renal Infection With Sars-Cov-2. J. Am. Soc Nephrol. 31, 1683–1687. doi: 10.1681/ASN.2020040432 32371536 PMC7460898

[B32] Ferrario C. M. Jessup J. Chappell M. C. Averill D. B. Brosnihan K. B. Tallant E. A.. (2005). Effect of Angiotensin-Converting Enzyme Inhibition and Angiotensin II Receptor Blockers on Cardiac Angiotensin-Converting Enzyme 2. Circulation 111, 2605–2610. doi: 10.1161/CIRCULATIONAHA.104.510461 15897343

[B33] Fontana F. Cazzato S. Giovanella S. Ballestri M. Leonelli M. Mori G.. (2020). Oxalate Nephropathy Caused by Excessive Vitamin C Administration in 2 Patients With COVID-19. Kidney Int. Rep. 5, 1815–1822. doi: 10.1016/j.ekir.2020.07.008 32838081 PMC7363608

[B34] Fowler A. A. Syed A. A. Knowlson S. Sculthorpe R. Farthing D. DeWilde C.. (2014). Phase I Safety Trial of Intravenous Ascorbic Acid in Patients With Severe Sepsis. J. Transl. Med. 12, 1–10. doi: 10.1186/1479-5876-12-32 24484547 PMC3937164

[B35] Fyhrquist F. Saijonmaa O. (2008). Renin-Angiotensin System Revisited. J. Intern. Med. 264, 224–236. doi: 10.1111/j.1365-2796.2008.01981.x 18793332 PMC7166930

[B36] Gautier-Vargas G. Baldacini C. Benotmane I. Keller N. Caillard S. (2020). Rapid Resolution of Cytokine Release Syndrome and Favorable Clinical Course of Severe COVID-19 in a Kidney Transplant Recipient Treated With Tocilizumab. Kidney Int. 98, 508–509. doi: 10.1016/j.kint.2020.05.022 32505467 PMC7272169

[B37] Guan W. J. Ni Z. Y. Hu Y. Liang W. H. Ou C. Q. He J. X.. (2020). Clinical Characteristics of Coronavirus Disease 2019 in China. N. Engl. J. Med. 382, 1708–1720. doi: 10.1056/NEJMoa2002032 32109013 PMC7092819

[B38] Hamming I. Timens W. Bulthuis M. L. Lely A. T. Navis G. van Goor H. (2004). Tissue Distribution of ACE2 Protein, the Functional Receptor for SARS Coronavirus. A First Step in Understanding SARS Pathogenesis. J. Pathol. 203, 631–637. doi: 10.1002/path.1570 15141377 PMC7167720

[B39] He W. Liu X. Feng L. Xiong S. Li Y. Chen L.. (2020a). Impact of SARS-CoV-2 on Male Reproductive Health: A Review of the Literature on Male Reproductive Involvement in COVID-19. Front. Med. 7. doi: 10.3389/fmed.2020.594364 PMC7711165 33330557

[B40] He Q. Mok T. N. Yun L. He C. Li J. Pan J. (2020b). Single-Cell RNA Sequencing Analysis of Human Kidney Reveals the Presence of ACE2 Receptor: A Potential Pathway of COVID-19 Infection. Mol. Genet. Genomic Med. 8, 1–11. doi: 10.1002/mgg3.1442 PMC7435545 32744436

[B41] He Z. Zhao C. Dong Q. Zhuang H. Song S. Peng G.. (2005). Effects of Severe Acute Respiratory Syndrome (SARS) Coronavirus Infection on Peripheral Blood Lymphocytes and Their Subsets. Int. J. Infect. Dis. 9, 323–330. doi: 10.1016/j.ijid.2004.07.014 16095942 PMC7110876

[B42] Hirsch J. S. Ng J. H. Ross D. W. Sharma P. Shah H. H. Barnett R. L.. (2020). Acute Kidney Injury in Patients Hospitalized With COVID-19. Kidney Int. 98, 209–218. doi: 10.1016/j.kint.2020.05.006 32416116 PMC7229463

[B43] Hoffmann M. Kleine-Weber H. Schroeder S. Krüger N. Herrler T. Erichsen S.. (2020). SARS-CoV-2 Cell Entry Depends on ACE2 and TMPRSS2 and Is Blocked by a Clinically Proven Protease Inhibitor. Cell 181, 271–280.e8. doi: 10.1016/j.cell.2020.02.052 32142651 PMC7102627

[B44] Hsu C. M. Gupta S. Tighiouart H. Goyal N. Faugno A. J. Tariq A.. (2022). Kidney Recovery and Death in Critically Ill Patients With COVID-19–Associated Acute Kidney Injury Treated With Dialysis: The STOP-COVID Cohort Study. Am. J. Kidney Dis., 79, 404–416. doi: 10.1053/j.ajkd.2021.11.004 34871701 PMC8641974

[B45] Huang C. Wang Y. Li X. Ren L. Zhao J. Hu Y.. (2020). Clinical Features of Patients Infected With 2019 Novel Coronavirus in Wuhan, China. Lancet 395, 497–506. doi: 10.1016/S0140-6736(20)30183-5 31986264 PMC7159299

[B46] Hung A. M. Shah S. C. Bick A. G. Yu Z. Chen H.-C. Hunt C. M.. (2022). APOL1 Risk Variants, Acute Kidney Injury, and Death in Participants With African Ancestry Hospitalized With COVID-19 From the Million Veteran Program. JAMA Intern. Med. 182, 386–395. doi: 10.1001/jamainternmed.2021.8538 35089317 PMC8980930

[B47] Iadecola C. Anrather J. Kamel H. (2020). Effects of COVID-19 on the Nervous System. Cell 183, 16–27.e1. doi: 10.1016/j.cell.2020.08.028 32882182 PMC7437501

[B48] Iwasaki M. Saito J. Zhao H. Sakamoto A. Hirota K. Ma D. (2021). Inflammation Triggered by SARS-CoV-2 and ACE2 Augment Drives Multiple Organ Failure of Severe COVID-19: Molecular Mechanisms and Implications. Inflammation 44, 13–34. doi: 10.1007/s10753-020-01337-3 33029758 PMC7541099

[B49] Jedlowski P. M. Jedlowski M. F. (2022). Coronavirus Disease 2019-Associated Immunoglobulin A Vasculitis/Henoch–Schönlein Purpura: A Case Report and Review. J. Dermatol. 49, 190–196. doi: 10.1111/1346-8138.16211 34741345 PMC8652426

[B50] Jewell P. D. Bramham K. Galloway J. Post F. Norton S. Teo J.. (2021). COVID-19-Related Acute Kidney Injury; Incidence, Risk Factors and Outcomes in a Large UK Cohort. BMC Nephrol. 22, 1–11. doi: 10.1186/s12882-021-02557-x 34719384 PMC8557997

[B51] Jin G. Y. Jina L. L. Zheng J. He B. J. (2020). Advantages of Anti-Inflammatory Acupuncture in Treating Sepsis of Novel Coronavirus Pneumonia. World J. Tradit. Chin. Med. 6 (2). doi: 10.4103/wjtcm.wjtcm_12_20.

[B52] Karki R. Kanneganti T. D. (2021). The ‘Cytokine Storm’: Molecular Mechanisms and Therapeutic Prospects. Trends Immunol. 42, 681–705. doi: 10.1016/j.it.2021.06.001 34217595 PMC9310545

[B53] Karnik S. S. Unal H. Kemp J. R. Tirupula K. C. Eguchi S. Vanderheyden P.. (2015). International Union of Basic and Clinical Pharmacology. XCIX. Angiotensin Receptors: Interpreters of Pathophysiological Angiotensinergic Stimuli. Pharmacol. Rev. 67, 820. doi: 10.1124/pr.114.010454err 26315714 PMC4630565

[B54] Khismatullin R. R. Ponomareva A. A. Nagaswami C. Ivaeva R. A. Montone K. T. Weisel J. W.. (2021). Pathology of Lung-Specific Thrombosis and Inflammation in COVID-19. J. Thromb. Haemost. 19, 3062–3072. doi: 10.1111/jth.15532 34538029 PMC8646730

[B55] Kilis‐pstrusinska K. Akutko K. Braksator J. Dancewicz A. Grosman-dziewiszek P. Jamer T.. (2021). Kidney Dysfunction and its Progression in Patients Hospitalized Duo to Covid-19: Contribution to the Clinical Course and Outcomes. J. Clin. Med. 10 (23). doi: 10.3390/jcm10235522 PMC8658310 34884225

[B56] Liotta E. M. Batra A. Clark J. R. Shlobin N. A. Hoffman S. C. Orban Z. S.. (2020). Frequent Neurologic Manifestations and Encephalopathy-Associated Morbidity in Covid-19 Patients. Ann. Clin. Transl. Neurol. 7, 2221–2230. doi: 10.1002/acn3.51210 33016619 PMC7664279

[B57] Liu J. Li S. Liu J. Liang B. Wang X. Wang H.. (2020). Longitudinal Characteristics of Lymphocyte Responses and Cytokine Profiles in the Peripheral Blood of SARS-CoV-2 Infected Patients. EBioMedicine 55, 102763. doi: 10.1016/j.ebiom.2020.102763 32361250 PMC7165294

[B58] Lucas C. Wong P. Klein J. Castro T. B. R. Silva J. Sundaram M.. (2020). Longitudinal Analyses Reveal Immunological Misfiring in Severe COVID-19. Nature 584, 463–469. doi: 10.1038/s41586-020-2588-y 32717743 PMC7477538

[B59] Macor P. Durigutto P. Mangogna A. Bussani R. De Maso L. D’errico S.. (2021). Multiple-Organ Complement Deposition on Vascular Endothelium in COVID-19 Patients. Biomedicines 9, 1–15. doi: 10.3390/biomedicines9081003 PMC8394811 34440207

[B60] Marques F. Gameiro J. Oliveira J. Fonseca J. A. Duarte I. Bernardo J.. (2021). Acute Kidney Disease and Mortality in Acute Kidney Injury Patients With Covid-19. J. Clin. Med. 10 (19). doi: 10.3390/jcm10194599 PMC8509682 34640618

[B61] Matsuyama S. Nao N. Shirato K. Kawase M. Saito S. Takayama I.. (2020). Enhanced Isolation of SARS-CoV-2 by TMPRSS2- Expressing Cells. Proc. Natl. Acad. Sci. U. S. A. 117, 7001–7003. doi: 10.1073/pnas.2002589117 32165541 PMC7132130

[B62] McMillan P. Dexhiemer T. Neubig R. R. Uhal B. D. (2021). COVID-19—A Theory of Autoimmunity Against ACE-2 Explained. Front. Immunol. 12. doi: 10.3389/fimmu.2021.582166 PMC8021777 33833750

[B63] Merad M. Martin J. C. (2020). Pathological Inflammation in Patients With COVID-19: A Key Role for Monocytes and Macrophages. Nat. Rev. Immunol. 20, 355–362. doi: 10.1038/s41577-020-0331-4 32376901 PMC7201395

[B64] Nadim M. K. Forni L. G. Mehta R. L. Connor M. J. Liu K. D. Ostermann M.. (2020). COVID-19-Associated Acute Kidney Injury: Consensus Report of the 25th Acute Disease Quality Initiative (ADQI) Workgroup. Nat. Rev. Nephrol. 16, 747–764. doi: 10.1038/s41581-020-00356-5 33060844 PMC7561246

[B65] Naicker S. Yang C. W. Hwang S. J. Liu B. C. Chen J. H. Jha V. (2020). The Novel Coronavirus 2019 Epidemic and Kidneys. Kidney Int. 97, 824–828. doi: 10.1016/j.kint.2020.03.001 32204907 PMC7133222

[B66] Na K. R. Kim H. R. Ham Y. Choi D. E. Lee K. W. Moon J. Y.. (2020). Acute Kidney Injury and Kidney Damage in COVID-19 Patients. J. Korean Med. Sci. 35, 1–9. doi: 10.3346/jkms.2020.35.e257 PMC7371456 32686373

[B67] Nataraj C. Oliverio M. I. Mannon R. B. Mannon P. J. Audoly L. P. Amuchastegui C. S.. (1999). Angiotensin II Regulates Cellular Immune Responses. J. Clin. Investig. 104, 1693–1701. doi: 10.1172/JCI7451 10606623 PMC409880

[B68] Noris M. Benigni A. Remuzzi G. (2020). The Case of Complement Activation in COVID-19 Multiorgan Impact. Kidney Int. 98, 314–322. doi: 10.1016/j.kint.2020.05.013 32461141 PMC7246017

[B69] Panitchote A. Mehkri O. Hasting A. Hanane T. Demirjian S. Torbic H.. (2019). Factors Associated With Acute Kidney Injury in Acute Respiratory Distress Syndrome. Ann. Intensive Care 9 (1), 74. doi: 10.1186/s13613-019-0552-5 31264042 PMC6603088

[B70] Pan X.-W. Xu D. Zhang H. Zhou W. Wang L. Cui X.-G. (2020). Identification of a Potential Mechanism of Acute Kidney Injury During the COVID-19 Outbreak: A Study Based on Single-Cell Transcriptome Analysis. Intensive Care Med. 46, 1114–1116. doi: 10.1007/s00134-020-06026-1 32236644 PMC7106051

[B71] Procaccini F. L. Alcázar Arroyo R. Albalate Ramón M. Torres Aguilera E. Martín Navarro J. Ryan Murua P.. (2021). Acute Kidney Injury in 3182 Patients Admitted With COVID-19: A Single-Center, Retrospective, Case–Control Study. Clin. Kidney J. 14, 1557–1569. doi: 10.1093/ckj/sfab021 34079618 PMC7929007

[B72] Qin C. Zhou L. Hu Z. Zhang S. Yang S. Tao Y.. (2020). Dysregulation of Immune Response in Patients With Coronavirus 2019 (COVID-19) in Wuhan, China. Clin. Infect. Dis. 71, 762–768. doi: 10.1093/cid/ciaa248 32161940 PMC7108125

[B73] Rahimzadeh H. Kazemian S. Rahbar M. Farrokhpour H. Montazeri M. Kafan S.. (2021). The Risk Factors and Clinical Outcomes Associated With Acute Kidney Injury in Patients With COVID-19: Data From a Large Cohort in Iran. Kidney Blood Press Res. 46, 620–628. doi: 10.1159/000517581 34315161 PMC8450864

[B74] Rahmani W. Chung H. Sinha S. Bui-Marinos M. P. Arora R. Jaffer A.. (2022). Attenuation of SARS-CoV-2 Infection by Losartan in Human Kidney Organoids. iScience 25, 103818. doi: 10.1016/j.isci.2022.103818 35106453 PMC8795780

[B75] Richardson S. Hirsch J. S. Narasimhan M. Crawford J. M. McGinn T. Davidson K. W.. (2020). Presenting Characteristics, Comorbidities, and Outcomes Among 5700 Patients Hospitalized With COVID-19 in the New York City Area. JAMA - J. Am. Med. Assoc. 323, 2052–2059. doi: 10.1001/jama.2020.6775 PMC7177629 32320003

[B76] Ronco C. Reis T. (2020). Kidney Involvement in COVID-19 and Rationale for Extracorporeal Therapies. Nat. Rev. Nephrol. 16, 308–310. doi: 10.1038/s41581-020-0284-7 32273593 PMC7144544

[B77] Ronco C. Reis T. Husain-Syed F. (2020). Management of Acute Kidney Injury in Patients With COVID-19. Lancet Respir. Med. 8, 738–742. doi: 10.1016/S2213-2600(20)30229-0 32416769 PMC7255232

[B78] Scarpioni R. Valsania T. Albertazzi V. Blanco V. DeAmicis S. Manini A.. (2021). Acute Kidney Injury, a Common and Severe Complication in Hospitalized Patients During the COVID-19 Pandemic. J. Nephrol. 34, 1019–1024. doi: 10.1007/s40620-021-01087-x 34146335 PMC8214067

[B79] Scrascia G. Rotunno C. Simone S. Montemurno E. Amorese L. Palo M.. (2017). Acute Kidney Injury in High-Risk Cardiac Surgery Patients: Roles of Inflammation and Coagulation. J. Cardiovasc. Med. 18 (5), 359–365. doi: 10.2459/JCM.0000000000000343 26657082

[B80] Shang J. Wan Y. Luo C. Ye G. Geng Q. Auerbach A.. (2020). Cell Entry Mechanisms of SARS-CoV-2. Proc. Natl. Acad. Sci. U. S. A. 117, 1–8. doi: 10.1073/pnas.2003138117 PMC7260975 32376634

[B81] Sindhu C. Prasad P. Elumalai R. Matcha J. (2022). Clinical Profile and Outcomes of COVID-19 Patients With Acute Kidney Injury: A Tertiary Centre Experience From South India. Clin. Exp. Nephrol. 26, 36–44. doi: 10.1007/s10157-021-02123-7 34401969 PMC8366740

[B82] Soleimani M. (2020). Acute Kidney Injury in Sars-Cov-2 Infection: Direct Effect of Virus on Kidney Proximal Tubule Cells. Int. J. Mol. Sci. 21. (9) doi: 10.3390/ijms21093275 PMC7247357 32380787

[B83] Strohbehn I. A. Zhao S. Seethapathy H. Lee M. Rusibamayila N. Allegretti A. S.. (2021). Acute Kidney Injury Incidence, Recovery, and Long-Term Kidney Outcomes Among Hospitalized Patients With COVID-19 and Influenza. Kidney Int. Rep. 6, 2565–2574. doi: 10.1016/j.ekir.2021.07.008 34307971 PMC8280679

[B84] Sullivan M. K. Lees J. S. Drake T. M. Docherty A. B. Oates G. Hardwick H. E. (2021). Acute Kidney Injury in Patients Hospitalised With COVID-19 From the ISARIC WHO CCP-UK Study: A Prospective, Multicentre Cohort Study Michael. Nephrol. Dial. Transplant. 27708, 1–19. doi: 10.1093/ndt/gfab303 PMC8788218 34661677

[B85] Su H. Yang M. Wan C. Yi L. X. Tang F. Zhu H. Y.. (2020). Renal Histopathological Analysis of 26 Postmortem Findings of Patients With COVID-19 in China. Kidney Int. 98, 219–227. doi: 10.1016/j.kint.2020.04.003 32327202 PMC7194105

[B86] Swol J. Lorusso R. (2020). Additive Treatment Considerations in COVID-19—The Clinician’s Perspective on Extracorporeal Adjunctive Purification Techniques. Artif. Organs 44, 918–925. doi: 10.1111/aor.13748 32516506 PMC7300593

[B87] Tang N. Li D. Wang X. Sun Z. (2020). Abnormal Coagulation Parameters are Associated With Poor Prognosis in Patients With Novel Coronavirus Pneumonia. J. Thromb. Haemost. 18, 844–847. doi: 10.1111/jth.14768 32073213 PMC7166509

[B88] Tan L. Wang Q. Zhang D. Ding J. Huang Q. Tang Y. Q.. (2020). Lymphopenia Predicts Disease Severity of COVID-19: A Descriptive and Predictive Study. Signal Transduction Targeting Ther. 5, 16–18. doi: 10.1038/s41392-020-0148-4 PMC7100419 32296069

[B89] Taverna G. Di Francesco S. Borroni E. M. Yiu D. Toniato E. Milanesi S.. (2021). The Kidney, COVID-19, and the Chemokine Network: An Intriguing Trio. Int. Urol. Nephrol. 53, 97–104. doi: 10.1007/s11255-020-02579-8 32720031 PMC7384276

[B90] Tay M. Z. Poh C. M. Rénia L. MacAry P. A. Ng L. F. P. (2020). The Trinity of COVID-19: Immunity, Inflammation and Intervention. Nat. Rev. Immunol. 20, 363–374. doi: 10.1038/s41577-020-0311-8 32346093 PMC7187672

[B91] Terpos E. Ntanasis-Stathopoulos I. Elalamy I. Kastritis E. Sergentanis T. N. Politou M.. (2020). Hematological Findings and Complications of COVID-19. Am. J. Hematol. 95, 834–847. doi: 10.1002/ajh.25829 32282949 PMC7262337

[B92] Tetlow S. Segiet-Swiecicka A. O’Sullivan R. O’Halloran S. Kalb K. Brathwaite-Shirley C.. (2021). ACE Inhibitors, Angiotensin Receptor Blockers and Endothelial Injury in COVID-19. J. Intern. Med. 289, 688–699. doi: 10.1111/joim.13202 33210357 PMC7753609

[B93] Tian S. Hu W. Niu L. Liu H. Xu H. Xiao S. Y. (2020). Pulmonary Pathology of Early-Phase 2019 Novel Coronavirus (COVID-19) Pneumonia in Two Patients With Lung Cancer. J. Thorac. Oncol. 15, 700–704. doi: 10.1016/j.jtho.2020.02.010 32114094 PMC7128866

[B94] Varga Z. Flammer A. J. Steiger P. Haberecker M. Andermatt R. Zinkernagel A. S.. (2020). Endothelial Cell Infection and Endotheliitis in COVID-19. Lancet 395, 1417–1418. doi: 10.1016/S0140-6736(20)30937-5 32325026 PMC7172722

[B95] Vickers C. Hales P. Kaushik V. Dick L. Gavin J. Tang J.. (2002). Hydrolysis of Biological Peptides by Human Angiotensin-Converting Enzyme-Related Carboxypeptidase. J. Biol. Chem. 277, 14838–14843. doi: 10.1074/jbc.M200581200 11815627

[B96] Walls A. C. Park Y. J. Tortorici M. A. Wall A. McGuire A. T. Veesler D. (2020). Structure, Function, and Antigenicity of the SARS-CoV-2 Spike Glycoprotein. Cell 181, 281–292.e6. doi: 10.1016/j.cell.2020.02.058 32155444 PMC7102599

[B97] Wan Y. I. Bien Z. Apea V. J. Orkin C. M. Dhairyawan R. Kirwan C. J.. (2021). Acute Kidney Injury in COVID-19: Multicentre Prospective Analysis of Registry Data. Clin. Kidney J. 14, 2356–2364. doi: 10.1093/ckj/sfab071 34751235 PMC8083651

[B98] Wang K. Chen W. Zhang Z. Deng Y. Lian J. Q. Du P.. (2020a). CD147-Spike Protein is a Novel Route for SARS-CoV-2 Infection to Host Cells. Signal Transduction Targeting Ther. 5, 1–10. doi: 10.1038/s41392-020-00426-x PMC7714896 33277466

[B99] Wang M. Li T. Qiao F. Wang L. Li C. Gong Y. (2020b). Coronavirus Disease 2019 Associated With Aggressive Neurological and Mental Abnormalities Confirmed Based on Cerebrospinal Fluid Antibodies: A Case Report. Med. (Baltimore). 99, e21428. doi: 10.1097/MD.0000000000021428 PMC7478433 32898993

[B100] Wang Y. Zhang D. Du G. Du R. Zhao J. Jin Y.. (2020c). Remdesivir in Adults With Severe COVID-19: A Randomised, Double-Blind, Placebo-Controlled, Multicentre Trial. Lancet 395, 1569–1578. doi: 10.1016/S0140-6736(20)31022-9 32423584 PMC7190303

[B101] Wan Y. Li J. Shen L. Zou Y. Lan P. (2020). Enteric Involvement in Hospitalised Patients With COVID-19 Outside Wuhan. Lancet Gastroenterol. Hepatol. 5, 534–535. doi: 10.1016/S2468-1253(20)30118-7 32304638 PMC7159861

[B102] Wichmann D. Sperhake J. P. Lütgehetmann M. Steurer S. Edler C. Heinemann A.. (2020). Autopsy Findings and Venous Thromboembolism in Patients With COVID-19: A Prospective Cohort Study. Ann. Intern. Med. 173, 268–277. doi: 10.7326/M20-2003 32374815 PMC7240772

[B103] Williams B. Baker A. Q. Gallacher B. Lodwick D. (1995). Angiotensin II Increases Vascular Permeability Factor Gene Expression by Human Vascular Smooth Muscle Cells. Hypertension 25, 913–917. doi: 10.1161/01.HYP.25.5.913 7737726

[B104] Winkler A. Zitt E. Sprenger-Mähr H. Soleiman A. Cejna M. Lhotta K. (2021). SARS-CoV-2 Infection and Recurrence of Anti-Glomerular Basement Disease: A Case Report. BMC Nephrol. 22, 1–5. doi: 10.1186/s12882-021-02275-4 33639869 PMC7914035

[B105] Yang X. Yu Y. Xu J. Shu H. Xia J. Liu H.. (2020). Clinical Course and Outcomes of Critically Ill Patients With SARS-CoV-2 Pneumonia in Wuhan, China: A Single-Centered, Retrospective, Observational Study. Lancet Respir. Med. 8, 475–481. doi: 10.1016/S2213-2600(20)30079-5 32105632 PMC7102538

[B106] Ye M. Wysocki J. William J. Soler M. J. Cokic I. Batlle D. (2006). Glomerular Localization and Expression of Angiotensin-Converting Enzyme 2 and Angiotensin-Converting Enzyme: Implications for Albuminuria in Diabetes. J. Am. Soc Nephrol. 17, 3067–3075. doi: 10.1681/ASN.2006050423 17021266

[B107] Zhang Y. Zheng N. Hao P. Cao Y. Zhong Y. (2005). A Molecular Docking Model of SARS-CoV S1 Protein in Complex With its Receptor, Human ACE2. Comput. Biol. Chem. 29, 254–257. doi: 10.1016/j.compbiolchem.2005.04.008 15979045 PMC7106554

[B108] Zheng M. Gao Y. Wang G. Song G. Liu S. Sun D.. (2020). Functional Exhaustion of Antiviral Lymphocytes in COVID-19 Patients. Cell. Mol. Immunol. 17, 533–535. doi: 10.1038/s41423-020-0402-2 32203188 PMC7091858

[B109] Zhou L. Niu Z. Jiang X. Zhang Z. Zheng Y. Wang Z.. (2020a). SARS-CoV-2 Targets by the pscRNA Profiling of ACE2, TMPRSS2 and Furin Proteases. iScience 23, 101744. doi: 10.1016/j.isci.2020.101744 33134888 PMC7591870

[B110] Zhou F. Yu T. Du R. Fan G. Cao B. (2020b). Clinical Course and Risk Factors for Mortality of Adult Inpatients With COVID-19 in Wuhan, China: A Retrospective Cohort Study. Lancet 395, 1054–1062. doi: 10.1016/S0140-6736(20)30566-3 32171076 PMC7270627

